# Dynamic association of PfEMP1 and KAHRP in knobs mediates cytoadherence during Plasmodium invasion

**DOI:** 10.1038/srep08617

**Published:** 2015-03-02

**Authors:** Akshay Kumar Ganguly, Priyatosh Ranjan, Ashutosh Kumar, Neel Sarovar Bhavesh

**Affiliations:** 1 International Centre for Genetic Engineering and Biotechnology (ICGEB), Aruna Asaf Ali Marg, New Delhi, India – 110 067; 2 Department of Biosciences and Bioengineering, Indian Institute of Technology (IIT), Bombay, Mumbai, India – 400 076

## Abstract

*Plasmodium falciparum* infected erythrocytes display membrane knobs that are essential for their adherence to vascular endothelia and for prevention of clearance by the spleen. The knob associated histidine rich protein (KAHRP) is indispensable to knob formation and has been implicated in the recruitment and tethering of *P. falciparum* erythrocyte membrane protein–1 (PfEMP1) by binding to its cytoplasmic domain termed VARC. However, the precise mechanism of interaction between KAHRP and VARC is not very well understood. Here we report that both the proteins co-localize to membrane knobs of *P. falciparum* infected erythrocytes and have identified four positively charged linear sequence motifs of high intrinsic mobility on KAHRP that interact electrostatically with VARC in solution to form a fuzzy complex. The current study provides molecular insight into interaction between KAHRP and VARC in solution that takes place at membrane knobs.

Malaria is a disease of global prevalence with ~207 million cases being reported in 2012 alone. Among its causative agents, *Plasmodium falciparum* is the most lethal of known malarial parasites, being responsible for about 98% cases in Africa and 65% cases globally^1^. The pathogenicity of *P. falciparum* is, in part, due to its property of avoiding splenic defenses by sequestration of infected erythrocytes in the microvasculature of organs; a phenomenon known as cytoadherence. This sequestration causes vascular blockage and is a direct cause of cerebral and placental malaria^2^,^3^. Sequestered erythrocytes are characterized by biomechanical changes in their cytoskeleton and cell surface that are crucial for the survival of the parasite^4^.

Cytoadhesion is mediated by the large (200–350 kDa), antigenically variant *P. falciparum* erythrocyte membrane protein-1 (PfEMP1), which can bind to host receptors on the surface of vascular endothelial cells^5^. Exported PfEMP1 is seen to localize to specific regions on the erythrocyte plasma membrane called knobs^6^,^7^,^8^. Knobs are cup shaped electron dense protrusions of the membrane that aid in anchoring PfEMP1. Past studies have shown that knobs are essential for cytoadherence under physiological flow conditions^9^. Inside the cell, knob primarily consists of an 80–105 kDa exported parasite protein called knob associated histidine-rich protein (KAHRP)^10^,^11^. The KAHRP primary structure^11^,^12^ consists of a signal sequence targeting the parasitophorous vacuole, a plasmodium export element, a histidine rich region (K1), a central lysine rich repeat region (K2) and a C-terminal repeat region (K3) (Fig 1). K1 and K2 can separately bind to the acidic terminal sequence (ATS) of PfEMP1 (termed VARC) located on the cytoplasmic face of plasma membrane of *P. falciparum*–infected erythrocytes^13^. Biochemical studies have shown that the N-terminal region of K2 of KAHRP (termed K2A) binds to the first 291 residues of VARC (comprising VARC A and VARC B) by a moderate affinity interaction that is pH dependent^14^,^15^.

The current study focuses on the interaction between K2A and VARC in solution and its implications on the mechanism of PfEMP1 tethering to knobs, using a combination of solution-state nuclear magnetic resonance (NMR) spectroscopy, calorimetry and immunofluorescence microscopy.

## Results

### K2A possesses two tandem repeats with overlapping resonance frequencies

**Figure 1:**
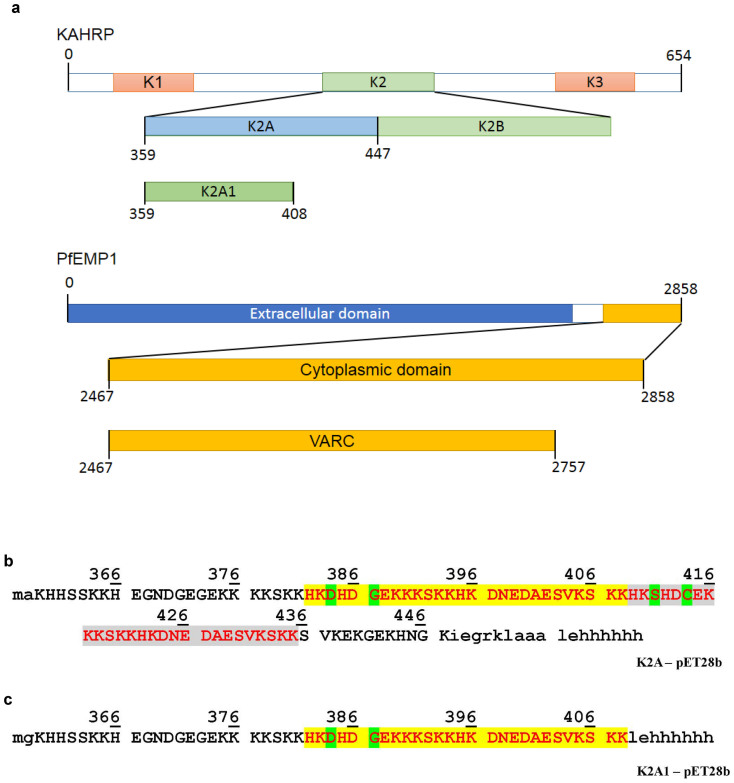
Domain architecture of K2A, K2A1 and VARC. (a) Schematic representations of KAHRP and PfEMP1 depicting cloned regions K2A, K2A1 and VARC. (b) Sequence of cloned K2A polypeptide showing tandem repeats (highlighted in yellow and grey). Amino acids highlighted in green represent the differences between the two repeats. Amino acids in lowercase are encoded by the vector pET28b. (c) Sequence of cloned K2A1 polypeptide, containing the first of two tandem repeats.

To gain molecular insight into the role of K2A, the central lysine-rich repeat region of KAHRP (Fig. 1a), it was over-expressed, purified and solution-state NMR spectroscopy was performed. The 2D [^15^N,^1^H] HSQC spectrum of K2A (Supplementary Fig. S1a) showed poor dispersion for the backbone amide resonances. During the course of obtaining backbone resonance assignments for K2A, it was observed that the chemical shift for a stretch of amino acids from positions H382 to K408 overlapped with those from positions H409 to K435 due to their being nearly perfect tandem repeats of each other (Fig. 1b) and hence, in similar chemical environments. The problem in obtaining sequence-specific resonance assignment posed by the tandem repeats of K2A was overcome by cloning the first (5′) repeat of K2A independently (termed K2A1) (Fig. 1c). The rationale for truncating K2A is corroborated by the fact that the interaction of K2A with VARC is largely electrostatic in nature^15^ and is unlikely to be altered on truncation, given that the theoretical isoelectric pI values of both K2A and K2A1 are identical (9.77) and both possess a net positive charge (+16.8 and +9.4, respectively) at pH 7. Full length K2A has a mean theoretical charge of +0.198 per residue while that for K2A1 was +0.188, ranging from values of −0.6 to +0.8. A comparison of the sequence-specific charge profiles of K2A and K2A1 (Supplementary Fig. S1b) showed an oscillating charge distribution along the length of both polypeptides, with discrete regions of high positive charge separated by negatively charged stretches. The similarity in charge distribution between the pair of positively charged tandem repeats suggested that only one of them would be sufficient for electrostatic interactions with acidic residues on VARC.

### K2A1 is an intrinsically disordered protein

**Figure 2:**
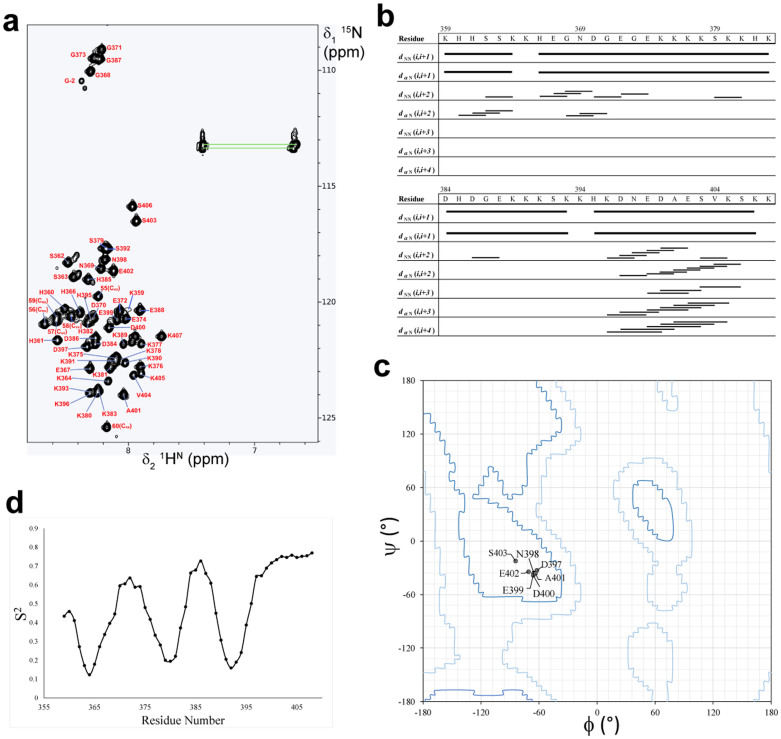
Biophysical analyses of K2A1 in solution. (a) 2D [^15^N,^1^H] HSQC spectrum of K2A1 recorded at 278 K at pH 6.2 on a Bruker *Avance* III 500 MHz spectrometer equipped with a cryogenic triple-resonance 5 mm TCI probe-head. Backbone amide resonance assignments are labeled. Resonances of extra residues derived from the plasmid vector on the C-terminus are labeled C_ex_ in parentheses. (b) NOE connectivity plot for K2A1 showing sequential (|i-j| = 1) and short to medium range (1 < |i-j| ≤ 4) H^N^ - H^N^ and H^N^ - H^α^ connectivities. (c) Ramachandran plot for residues D397 to S403, exhibiting *ϕ*, *ψ* torsion angle values approximating to that of a right – handed helix. (d) Residue specific model-free backbone order parameter (S^2^) values for K2A1 derived from observed chemical shifts showing backbone dynamics of K2A1.

Poor dispersion and narrow line-widths of backbone amide resonances in the 2D [^15^N,^1^H] HSQC spectrum of K2A1 revealed the presence of highly unstructured and flexible regions similar to K2A (Fig. 2a). All NMR spectra for K2A1 and K2A were measured at 278 K where backbone amide resonances were more dispersed and had better line-widths. A comparison of the 2D [^15^N,^1^H] HSQC spectra of K2A1 and K2A revealed an overlap of large number of backbone amide peaks despite the 48 residue difference in length between the recombinant proteins, further justifying our rationale for truncating K2A (Supplementary Fig. S2).

^1^H,^15^N and ^13^C backbone and side-chain resonances of K2A1 were assigned, except for the NHs of residues K355, K394 and K408. About ~92% of backbone and ~56% of side chain resonances were assigned unambiguously. Complete unambiguous side-chain resonance assignment was not possible due to the poor chemical shift dispersion of methylene and methyl protons as well as for side chain NH resonances that is expected for an intrinsically disordered polypeptide.

Automated NOE resonance assignments were performed using 3D ^13^C-edited [^1^H,^1^H]-NOESY and 3D ^15^N-edited [^1^H,^1^H]-NOESY spectra, yielding a total of 151 NOE distance restraints (2.52 per residue) and 257 dihedral restraints for distance geometry calculations. Medium (1 < |i-j| < 5) and long range (|i-j| > 4) NOEs constituted only 8.61% and 3.31% of observed NOEs respectively, the remainder being contributed by intra-residue (|i-j| = 0) and sequential (|i-j| = 1) NOEs. Secondary chemical shifts and the absence of mid- and long-range [^1^H,^1^H] NOEs clearly proved that K2A1 is an intrinsically disordered protein (IDP) at a physiological pH (Fig. 2b, Supplementary Fig. S3a). In a series of 2D [^15^N,^1^H] HSQC spectra measured for K2A1 from 278 K to 318 K in steps of 5 K, the dispersion of ^1^H^N^ resonances increased with decrease in temperature. Lowering of temperature to 278 K also resulted in the appearance of several backbone amide resonances along with a small increase in the chemical shift dispersion and average peak intensity (Supplementary Fig. S3b,c). This could be attributed to a decrease in amide proton exchange rates, possible stabilization of local structural elements and a reduction of conformational exchange at lower temperatures. This behavior is unique to IDPs and unfolded proteins^16^ and is opposite of what is expected for a globular protein wherein lower temperatures reduce the molecular tumbling rate, causing line broadening due to fast transverse relaxation rates (R_2_). Some studies have shown that IDPs have a propensity to form *α*-helices at low temperatures and sample a smaller conformational space^16^,^17^. Interestingly, the 3D ^15^N-edited [^1^H,^1^H] NOESY strips of residues D397 to S406 showed a number of short to medium range (1 < |i-j| < 4) H^N^–H^α^ and H^N^–H^N^ NOEs as compared to other residues in K2A1 (Supplementary Fig. S3d). Additionally, the backbone torsion angle values (*φ*, *ψ*) of D397 to S403 as derived from TALOS+ were found to fall in the region of the Ramachandran plot corresponding to a right-handed helix^18^ (Fig. 2c, Supplementary table S1), while the remainder were indicative of a backbone lacking in secondary structural elements.

We determined the backbone dynamics of K2A1 in terms of model-free order parameters (S^2^) from observed chemical shifts^19^, using TALOS+. The residue-wise backbone S^2^ values showed three regions of high local mobility (0 ≤ S^2^ ≤ 0.5) alternating with regions of relatively higher order (0.5 <S^2^ ≤ 1.0) along the length of the K2A1 polypeptide chain (Fig. 2d). The stretch of residues corresponding to the helix-like structural element was highly ordered (0.6 < S^2^ ≤ 0.8) relative to the rest of the polypeptide. Sequence analysis of this particular stretch revealed that it has a higher hydrophobicity (0.344 ± 0.092) as compared to full length K2A1 (0.195 ± 0.09). Given that past studies have linked side chain hydrophobicities to *α*-helical propensity and stability^20^,^21^,^22^, we were able to conclude that a transient helix-like structure exists in this stretch, which is populated at lower temperatures due to reduction in conformational exchange rate.

### K2A1 binds VARC in solution via four dynamic, positively charged, linear sequence motifs

**Figure 3:**
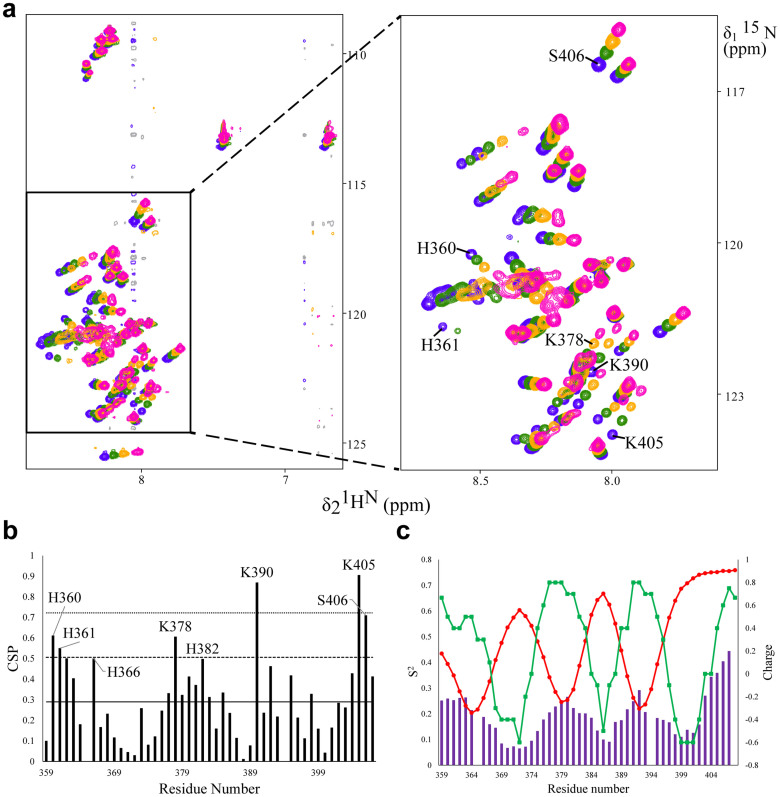
K2A1-VARC interaction in solution. (a) Overlay of 2D [^15^N,^1^H] HSQC spectra of free K2A1 (blue) versus 1:1 (green), 1:2.5 (ochre) and 1:5 (pink) molar ratios of ^15^N-labeled K2A1 to unlabeled VARC. Demarcated residues are those undergoing significant perturbation. (b)^1^H^N^ and ^15^N CSPs for K2A1 upon binding to 5 molar equivalents of VARC. The horizontal lines represent the mean (-), mean plus one standard deviation (--) and mean plus two standard deviations (….). (c) Comparison of perturbation, charge and S^2^. Overlay of chemical shift perturbations of K2A1 on addition of VARC (purple bars), backbone order parameter S^2^ of free K2A1 (red dots) and charge profile of free K2A1 (green squares). All data were smoothed using a moving average of 5 to account for nearest neighbor effects. Correlations between the variables are given in Supplementary table S2.

In order to identify the interacting region on K2A1, it was next titrated against increasing VARC concentrations at 278 K and at very low ionic strengths (0.013 M). The residues involved in the interaction between K2A1 and VARC were mapped (Fig. 3a,b) using the chemical shift perturbation (CSP) in backbone amide chemical shifts of K2A1 upon interaction with VARC. The line-widths in 2D [^15^N,^1^H] HSQC spectra of K2A1 remain constant up to a molar ratio of 1:5, indicating an initial moderate to weak binding in the fast exchange regime^23^. Line broadening was observed on saturation with VARC after a molar ratio of 1:5 up to a molar ratio of 1:10 (Supplementary Fig. S4a). This is possibly due to a cooperative increase in affinity between the two proteins leading to the formation of a large complex having a very fast transverse relaxation rate (R_2_)^24^. No significant changes were observed in the control 2D [^15^N,^1^H] HSQC spectra of K2A1 titrated against increasing concentrations of bovine serum albumin (BSA) and human α-synuclein, thus discounting crowding effects and non-specific electrostatic interactions as contributing factors to chemical shift perturbation (Supplementary Fig. S4b,c). Amide chemical shift perturbation profile for the complex observed at 150 mM NaCl concentration (Supplementary Fig. S5) indicated the stability of the complex at a physiological salt concentration. However few residues showed small chemical shift perturbations and changes in amide line widths as compared to the complex at 0 mM NaCl, indicative of possible changes in exchange rate, while bulk K2A1 remains in the VARC-bound state. The spectra of the complex at no salt were used for further analysis as they had better line-widths.

Residues H360, H361, K378, H382, K390, K405 and S406 showed higher CSPs (greater than one standard deviation from the mean) relative to other residues and are likely to be involved in the interaction (Fig. 3b). Lysine (pI = 9.74) and histidine (pI = 7.59) residues have a net positive charge at pH 6.2 and hence, are most likely to interact with acidic residues on VARC. A global observation of the perturbation profile showed four distinct regions on the K2A1 polypeptide (K359-D370 (region 1), G373-E388 (region 2), K390-D400 (region 3) and A401-K407 (region 4)) that are involved in the interaction.

An interesting correlation was observed between CSPs, sequence specific charge and order parameter (S^2^) of K2A1. Regions experiencing higher CSPs have net positive charge and are more flexible (low values of S^2^) (Fig. 3c). All four interacting regions showed a significant (*p < 0.05*) positive correlation between CSPs and charge and a significant (*p < 0.05*) negative correlation between CSPs and S^2^ (Supplementary table S2). Barring region 4, all regions also showed a significant (*p < 0.05*) negative correlation between charge and S^2^. The direct implication of these observations was that the probability of a residue on K2A1 binding to VARC depends on its charge, the charge distribution in its immediate neighborhood and the local backbone dynamics. For instance, the tetra-lysine stretch from K375 to K378, showed a gradually increasing CSP from K375 to K378, rather than equal values for all four residues. This is partially due to the presence of a negatively charged stretch of amino acids (D370 to E374) at the N-terminal of K375 that conferred an effective charge of +0.2 on K375. Such instances of ‘nearest-neighbor’ effects were also observed for several serine residues that showed higher CSPs compared to other serines, owing to their being sandwiched between lysine residues (K378-S379-K380, K391-S392-K393 and K405-S406-K407). It might be possible that serines also contribute to binding by forming H-bonds.

Regions 1, 2 and 3 showed more backbone flexibility at their centers as compared to their respective edges, which would likely aid in their electrostatic binding to VARC by allowing a wider range of conformational sampling with exchanges occurring at sub-microsecond time-scales. These three regions would therefore, resemble ‘cationic clouds’ in solution, bridged by relatively less mobile negatively charged stretches. However, region 4 showed the largest CSPs despite being the most rigid segment of K2A1, indicating a possible role of the transiently structured region (D397-S403) in enhancing binding affinity.

In contrast to free K2A1, dispersion of backbone amide proton chemical shift did not increase for the complex (Supplementary Fig. S6). On the contrary, the dispersion decreased at molar concentrations of VARC exceeding that of K2A1, reaching a minima of ~0.7 ppm after a molar ratio of 1:5 (K2A1 to VARC). Since backbone amide proton chemical shift dispersion is a sensitive measure of secondary structures in IDPs^25^, one can assume that little or no disorder-to-order transition occurs for K2A1 upon binding to VARC. Instead, the partially structured state that was observed for K2A1 at 278 K was lost upon binding and it had the signature of a more coil-like global configuration. This departure from the classical ‘folding upon binding’ paradigm has been recently explored in cases of complexes involving IDPs and globular proteins with disordered regions and membrane proteins^26^. Moreover, several interactions between IDP pairs have also been found to remain disordered upon binding^27^,^28^,^29^.

**Figure 4:**
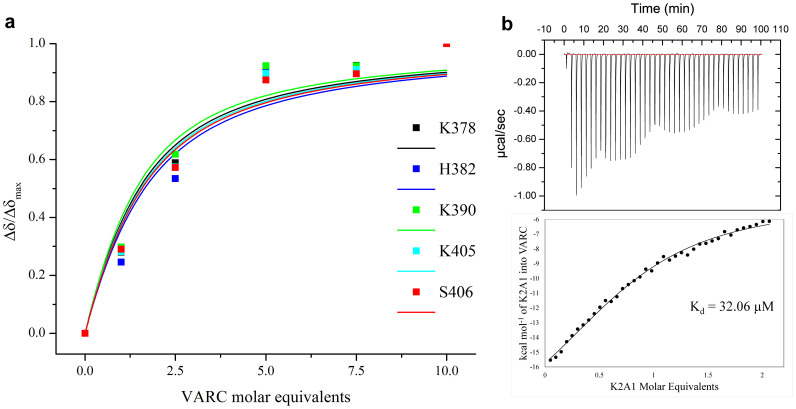
Binding affinities within the K2A1-VARC complex. (a) Non-linear least squares fit to eq. 4 of five resonances each for five representative residues having CSPs higher than mean plus 1σ. Dissociation constants and fitting statistics are tabulated in Supplementary table S3. (b) ITC isotherm of K2A1 binding on VARC. (Kd values for a four-site binding were 9.8 μM, 10.3 μM, 10.1 μM and 9.6 μM. Apparent global Kd was 32.06 μM.)

The observation of chemical shift perturbations upon titrating K2A1 against VARC necessitated the quantification of the strength of the interaction. We extracted dissociation constants for all residues that showed backbone amide perturbations greater than one standard deviation from the mean. Of these, fitted curves and dissociation constants of K378, H382, K390, K405 and S406 are shown as representatives (Fig. 4a, Supplementary Table S3). Within the limits of experimental error, the values for these residues were found to range between 7 and 15 μM that is in agreement with previously reported values^14^. We performed isothermal titration calorimetry (ITC) by titrating VARC against K2A1 at 298 K in a buffer of minimal ionic strength (0.013 M), so as to be able to observe the minutest of electrostatic interactions between oppositely charged species, as also to minimize charge shielding of amino acids by buffer counter ions. The pH of the interaction was buffered at 6.9 as the charge difference between K2A1 and VARC is likely to be highest at intermediate pH values between their respective theoretical isoelectric points (9.66 and 4.85, respectively). Assuming that four similar negatively charged regions on VARC would match the four interacting regions on K2A1, we fitted the ITC thermogram to a four site-binding model (Figure 4b). While the net stoichiometry of K2A1 and VARC in the complex remains unclear, the dissociation constants obtained from ITC are in agreement with those obtained for strongly interacting residues on K2A1. These values are similar to values reported in an earlier study, which had shown that full length K2A binds to various subdomains of VARC with K_d_ values ranging from 1.5 to 32 μM^14^. The average dissociation constant for the reaction was found to be 32.06 μM, indicative of a moderate to weak affinity binding as a whole, consistent with the narrow resonances obtained in the initial titration steps carried out using NMR.

### KAHRP and PfEMP1 co-localize in membrane knobs

**Figure 5:**
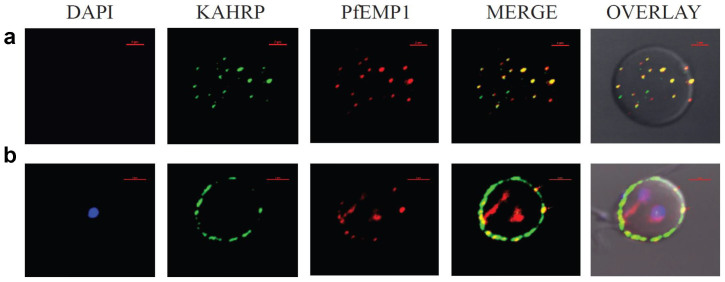
Immunofluorescence based co-localization of KAHRP (using anti-K2A1 antibodies) and PfEMP1 (using anti-VARC antibodies) in *P. falciparum* infected erythrocytes. Confocal laser scanning microscopy images of iRBCs showing individual images of DAPI stained parasite nuclei (blue), KAHRP (green), PfEMP1 (red), merged images of KAHRP and PfEMP1 and an overlay of the above on a DIC (differential interference contrast) image of the iRBC. Co-localizations are seen in yellow (red arrows). The red bar in each image corresponds to a scale of 2 μm. (a) Optical slice of the plasma membrane of a trophozoite infected RBC, showing extensive co-localization. (b) Cross-section of a trophozoite-infected RBC exhibiting co-localization on RBC membrane.

Previous *in vivo* studies have independently localized KAHRP and PfEMP1 to knobs using polyclonal antibodies raised against the full-length proteins^7^,^10^,^11^,^30^. However, a simultaneous probing of the two proteins has been lacking to date. In order to investigate the physical association of KAHRP and VARC inside the infected RBCs we used anti-K2A1 and anti-VARC antibodies to visualize KAHRP and PfEMP1. Interestingly, confocal laser scanning microscopy (CLSM) of *P. falciparum* infected erythrocytes showed the co-localization of KAHRP and PfEMP1 to the erythrocyte membrane (Fig. 5a, b). The co-localization was seen in several trophozoite infected RBCs, including early stage trophozoites with little hemozoin. No fluorescence was observed in the uninfected erythrocyte controls, eliminating cross-reactivity of the antibodies with erythrocyte components.

The degree of co-localization of the two fluorophores AlexaFluor488 and AlexaFluor594 are explained using Pearson's correlation and its coefficients c1 and c2^31^ (Supplementary table S4).

By combining the sensitive technique of fluorescence and the resolution offered by confocal laser scanning microscopy, an accurate measurement of co-localization could be made by statistically determining the degree of overlap between the fluorophores. As the Pearson's correlation fell between 0.6–0.8, it could be assumed that the localization of KAHRP (green) and PfEMP1 (red) was not completely mutually inclusive. This was because the export of PfEMP1 to the erythrocyte membrane is not dependent solely on KAHRP^30^,^32^. However, in regions where co-localization did occur, the coefficients c1 and c2 were in the range 0.97–1.0, implying a near perfect overlap at the optical resolution being used. Together with the NMR and calorimetric titration these immunofluorescence studies provide an *in vivo* evidence of co-localization of KAHRP and PfEMP1 (VARC) at the erythrocyte membrane.

## Discussion

The interactions between several parasite proteins that govern the related phenomena of knob formation and cytoadherence of *P. falciparum* infected erythrocytes (iRBCs) to endothelial cells have been extensively studied to date. Previous studies have shown that the knob associated histidine rich protein (KAHRP) is indispensable to knob formation in iRBCs^10^,^11^ and that mutant parasites with the KAHRP gene knocked out are unable to adhere to vascular endothelium under the shear stress imposed by physiological flow conditions^9^,^33^. Moreover, despite binding to the spectrin-actin junction of the erythrocyte cytoskeleton with a high affinity *via* its cytoplasmic domain, PfEMP1 requires KAHRP for additional stability^34^,^35^. Membrane expression of PfEMP1 is also impaired in KAHRP^−^ mutants, implying a role of KAHRP in recruitment of PfEMP1 molecules also^9^,^32^. Recent studies using immobilized protein assays have delineated the interaction between domains of KAHRP and PfEMP1^13^,^14^,^36^ to show that the central lysine rich repeat region of KAHRP, termed K2A, interacts with VARC with a moderate affinity electrostatic binding^15^. However, the absence of any detectable binding of these two domains in solution has been speculated upon in a recent study^37^. Our findings constitute the first report of the interaction between K2A and VARC in solution and its structural implications in the localization of PfEMP1 to knobs, using a combination of solution-state nuclear magnetic resonance spectroscopy, calorimetry and microscopic techniques.

K2A domain contains repeats of charged residues and is predicted to be a highly disordered domain^38^. Analysis of the 2D [^15^N,^1^H] HSQC spectrum of K2A, along with its abnormal electrophoretic migration patterns and presence of highly positively charged low complexity regions revealed it to be an intrinsically disordered protein (IDP) at physiological pH. VARC on the other hand, possesses a structured *α*-helical core surrounded by regions of high intrinsic disorder that shows little or no propensity for secondary structure formation under physiological conditions^37^.

Consolidation of data obtained from solution NMR studies and calorimetry enable us to arrive at a consensus model for the K2A1-VARC interaction and its implication in the tethering of PfEMP1 to membrane knobs. Long-range electrostatic attractive forces between four positively charged linear sequence motifs on K2A1 and anionic patches on VARC initiate complex formation. The inherent dynamism of the first three form ‘cationic clouds’ on K2A1 that provide the conformational variability required to increase chances of attaining binding favorable states. The binding of these three regions probably leads to loss of transient structural elements on K2A1, causing it to attain an extended coil-like configuration on the VARC surface. This frees the more rigid region 4 to participate in a final binding step. Given the line broadening observed on saturating K2A1 with VARC, the binding of K2A1 to VARC most likely undergoes a cooperative increase in affinity followed by a finite oligomerization till a larger structure is formed. The oligomerization process is likely to be driven by ionic cross-linking mediated by K2A1, which, *in vivo*, would cause the characteristic clustering of PfEMP1 on knobs. However, the four focal points of association are at best, held together by a moderate affinity interaction, resulting in a dynamic equilibrium between bound and unbound states. Such ‘fuzzy complexes’ have been described previously using the polyelectrostatic model, wherein multiple linear binding motifs constitute a dynamic ensemble of binding sites on a relatively ordered interacting partner^39^. However, the KAHRP-VARC complex as a whole may not be completely disordered and dynamic *in vivo*, in light of a recent finding^40^ that has shown the localization of a parasite encoded chaperone protein KAHsp40 to knobs, which may be instrumental in the stabilization and folding of the IDPs present therein.

In conclusion, the study of the phenomenon of cytoadherence has been hampered by the lack of biophysical and structural information on the macromolecular complexes that make up the knob. Given the presence of several low-complexity and unstructured regions in its constituent proteins, these complexes are not amenable to conventional methods of determining atomic resolution structures. Our study of the interaction between K2A1 and VARC corroborate the data previously available through immobilized assays^13^,^14^,^36^ and also provided a more detailed understanding of the mechanism of PfEMP1 tethering to membrane knobs. Based on evidence gathered from solution NMR and calorimetry we were able to conclude that K2A1 interacts with VARC in solution *via* four positively charged dynamic binding regions to form a disordered and dynamic complex. Oligomerization of this complex is probably due to cross-linking mediated by K2A1 forming the basis of accumulation of PfEMP1 molecules on knobs. The electrostatic nature of this interaction is largely due to long-range attractive forces between positively charged lysine and histidine residues on K2A1 and acidic residues on VARC. This knowledge would be indispensable in the screening of small molecules such as halogenated derivatives of lysine and histidine or cationic peptides that may be used to disrupt knob association of PfEMP1. From an *in vivo* point of view, the complex formation was put in perspective by our demonstration of the subcellular co-localization of the full-length proteins KAHRP and PfEMP1 in erythrocytes infected with trophozoite-stage *P. falciparum*. Our study thus provides a molecular glimpse into the tethering of PfEMP1 to knobs by KAHRP and may act as a seed for more detailed structural studies involving other domains of KAHRP, VARC and the erythrocyte cytoskeleton.

## Methods

### Molecular cloning and sequence analysis of VARC, K2A and K2A1

Expression clones of VARC domain of PfEMP1 (7399–8271 bp) encoding for amino acids 2467–2757 and the K2A domain of KAHRP (1075–1341 bp) encoding for amino acids 359–447 were obtained as a kind gift from Dr. Amit Sharma. Both were cloned in pET28b vector. The DNA fragment corresponding to K2A1 (1075–1224 bp of KAHRP, amino acid residues 359–408) was PCR amplified from the pET28b-K2A vector construct. The major amplification product of 177 base pairs was cloned into pET28b vector along with a C-terminal hexa-Histidine tag. The clonings were confirmed by DNA sequencing (Macrogen, Inc., Seoul, South Korea). The vector constructs were used to transform *Escherichia coli* BL21 (*DE3*) CodonPlus cells for protein over-expression. Phusion polymerase, restriction enzymes and T4 DNA Ligase were purchased from New England Biolabs. The plasmid vector pET28b and *E. coli* BL21 (*DE3*) CodonPlus cells were obtained from Novagen.

The theoretical isoelectric pH values of the proteins were determined using the ‘ProtParam’ tool on the ExPASy^41^ web server (http://web.expasy.org/protparam). Hydrophobicities were calculated using Kyte and Doolittle method^42^ in the ‘ProtScale’ tool (http://web.expasy.org/protscale/) with a window of 5 amino acids, normalized to values between 0 and 1. Residue level charge and net protein charge were calculated using the ‘charge’ tool on the EMBOSS web server (http://emboss.bioinformatics.nl), wherein lysine and arginine are assigned a net charge of +1 each; histidine, a net charge of +0.5 and aspartate and glutamate are each assigned a net charge of −1 at pH 7.0. The remaining amino acids are assigned a net charge of zero.

### Protein preparation and generation of antibodies

Unlabeled, uniformly ^15^N-labeled and [^15^N,^13^C]-labeled K2A and K2A1 were over-expressed in *E. coli* BL21(*DE3*) CodonPlus strain in M9 minimal media containing ^15^NH_4_Cl and ^13^C-glucose as the sole nitrogen and carbon sources respectively. Unlabeled VARC was similarly over-expressed in Luria-Bertani medium (USB chemicals). The recombinant proteins were expressed in soluble form. All protein purifications were carried out using Qiagen Ni-nitrilo triacetic acid (Ni-NTA) gravity flow columns, followed by gel permeation chromatography (GPC) on a GE 16/60 HiLoad Superdex 75 column to obtain samples of approximately >95% homogeneity. Sample purity was checked on SDS-PAGE gels. Eluted fractions were concentrated using centrifugal filters (Molecular weight cut-off of 3 kDa). K2A and K2A1 were concentrated up to 5–6 mg/ml (0.5–0.9 mM for K2A1 and K2A) while VARC was concentrated up to 1–2 mg/ml (0.05–0.07 mM). Given the absence of aromatic residues in K2A1, bicinchoninic acid test (at 40°C)^43^ validated by PULCON NMR technique^44^,^45^ were employed to determine the protein concentration. Protease inhibitor cocktails (Roche) and pepstatin were added in all protein preparations to prevent proteolytic degradation. Final preparations were divided into aliquots, flash frozen in liquid nitrogen and stored at −20°C. Given the high sensitivity to proteolysis for VARC, fresh aliquots were used for each experiment, with no more than one freeze/thaw cycle.

Generation of antibodies was carried out in rabbit (VARC) and mouse (K2A1) immunized against 90–95% pure protein samples. Polyclonal antibodies (IgG) specific for the respective protein were purified from antisera by antigen affinity chromatography and used for immunofluorescence experiments. Antibody preparations were procured from Bangalore Genei, India.

### NMR sample preparation

K2A1 sample was prepared for NMR using protein preparations concentrated to 0.5–0.7 mM in 10 mM sodium phosphate buffer, pH 6.2, containing 50 mM NaCl, 5 mM DTT, 5% D_2_O (*v/v*) and 0.01% NaN_3_ (*w/v*). NMR titrations of K2A1 against VARC were carried out in 10 mM sodium phosphate buffer, pH 6.2, containing 5 mM DTT, 5% D_2_O (*v/v*) and 0.01% NaN_3_ (*w/v*), without any additional salts. Samples also contained protease inhibitor cocktails (Roche) and pepstatin. Traces of 4,4-dimethyl-4-silapentane-1-sulfonic acid (DSS) were added as an internal reference.

### NMR data acquisition, processing and analysis

All experiments were carried out on Bruker *Avance* III spectrometers equipped with 5 mm cryogenic triple-resonance TCI probes, operating at field strengths (^1^H Larmor frequency) of 500 and 700 MHz. Temperature optimization of backbone ^1^H^N^ chemical shift dispersion and resonance line-widths was carried out by acquiring a series of 2D [^15^N,^1^H] HSQC spectra at temperatures varying from 278 K to 318 K, in steps of 5 K. For the purpose of resonance assignments, a set of standard double and triple resonance spectra namely 2D [^15^N,^1^H]-HSQC, 2D ^13^C′-detected NCO, 2D ^13^C′-detected CACO, 3D HNCA, 3D CBCAcoNH, 3D HNCACB, 3D HNCO, 3D CcccoNH, 3D HcccoNH, 2D [^13^C,^1^H]-HSQC, 3D ^15^N-edited [^1^H,^1^H]-NOESY and 3D ^13^C-edited [^1^H,^1^H]-NOESY (NOESY mixing time: 100 ms) were measured at 278 K. Topspin 2.1 (Bruker AG) software was used for acquisition, Fourier transformation and processing of time-domain data. ^13^C and ^15^N shifts referenced indirectly to the DSS methyl proton resonance at 0 ppm in all spectra. Backbone and side-chain resonances were assigned manually using Computer Aided Resonance Assignment (CARA)^46^. Automated NOE peak assignment and chemical shift validation were carried out using UNIO '10^47^,^48^. Calculation of torsion angle dynamics and distance geometries was attempted by simulated annealing of 10,000 steps in CYANA 3.0 using 7 iterative cycles. Dihedral angle values (*φ*, *ψ*) and model-free order parameters (S^2^) were derived from observed chemical shifts using TALOS+^19^,^49^ (http://spin.niddk.nih.gov/bax/nmrserver/talos/).

### NMR titrations of K2A1 against VARC

A series of 2D [^15^N,^1^H] HSQC spectra of ^15^N-labeled K2A1 were acquired at 278 K with an increasing concentration of unlabeled VARC up to saturation. 1:1, 1:2.5, 1:5, 1:7.5 and 1:10 molar ratios of K2A1 to VARC were used. K2A1 concentration was reduced to 10 μM to accommodate the higher (up to 1:10) molar ratios, given that VARC could be concentrated to a maximum of 3.4 mg/ml (100 μM) without aggregation. Correspondingly, number of scans used in the 2D [^15^N,^1^H] HSQC spectra was increased to 512 to obtain good signal-to-noise. We measured a series of 2D [^15^N,^1^H] HSQC spectra of the 1:5 K2A1-VARC complex at 20 mM, 50 mM, 100 mM and 150 mM NaCl to test the stability of the complex under higher ionic strengths. As controls,^15^N-labeled K2A1 was titrated against bovine serum albumin and human α-synuclein (1:0.5, 1:1, 1:2 and 1:5 molar ratios). Integrity of the sample was verified before and after each acquisition using SDS-PAGE. Owing to the proteolytic degradation of VARC after 4–5 hours in solution, we were unable to acquire lengthy triple resonance spectra or obtain dynamics information for K2A1 in the bound form. Perturbation in the backbone amide ^1^H^N^ and ^15^N chemical shifts (Δδ) was calculated using the formula 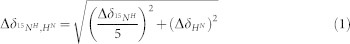
Where Δ*δ*(^1^H^N^) and Δ*δ*(^15^N) are the changes in backbone amide chemical shifts for ^1^H^N^ and ^15^N respectively, from free K2A1. CSPs for each residue were calculated for 1:1, 1:2.5 and 1:5 molar ratios of K2A1 to VARC. Higher molar ratios (1:7.5 and 1:10) had missing resonances in their HSQC spectra due to line broadening and were not included.

Pearson correlation coefficients between perturbations, charge and S^2^ (referred to as variables for simplicity) were calculated using a moving average with a window of 5 amino acids for each of the three variables, to account for nearest neighbor effects. The averaged value (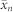) of a variable *x* (CSP, charge or S^2^) for the *n*th residue was calculated as: 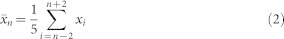
The Pearson correlation coefficients (r_x,y_) were computed using the formula:
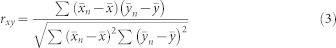
Where 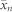 and 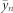 are averaged values of two variables *x* and *y* for the *n*th residue as calculated using eq. 2 while 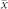 and 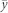 are the means of the corresponding variables across the entire range of residues being tested. The *P*-values for correlation coefficients were derived at a 95% confidence level using *n*-2 degrees of freedom. Dissociation constants were derived from amide chemical shift perturbation values^50^ by non-linear least squares fitting of five resonances to the equation
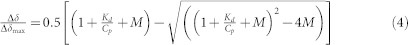
Where Δδ is the normalized amide chemical shift perturbation calculated in eq. 1, Δδ_max_ is the normalized amide chemical shift perturbation between free and VARC – saturated K2A1, K_d_ is the dissociation constant, C_p_ is the concentration of labeled K2A1 (10 μM) and M is the molar ratio of VARC to K2A1. All calculations and plotting of graphs were carried out using OriginPro v8.6, SigmaPlot v12 and Microsoft Excel 2013.

### Isothermal titration calorimetry

Isothermal titrations of VARC against K2A1 were performed on a GE MicroCal ITC_200_ calorimeter to obtain thermodynamic parameters of the interaction. Both proteins were prepared for titration by extensively dialysing against 10 mM sodium phosphate buffer, pH 6.9 containing 5 mM DTT, without any additional salts. 0.7 mM K2A1 was used as the syringe solution and 0.07 mM VARC was added to the ITC cell. Dilution effects were negated by titrating K2A1 against the buffer solution and subtracting the resultant isotherm from the experimental data. The titrations were carried out at 298 K over 40 injections of 1 μL each, with a time gap of 240 s between each injection, with constant stirring at 1000 rpm to ensure homogenous mixing. Data analyses and peak integration were carried out using Origin 7 software. To obtain a binding model consistent with the four binding regions of K2A1, heats were fitted to a sequential four site model, assuming that each one to one association of K2A1 and VARC occurs through attachment of four sites. For determination of a global dissociation constant, non-linear curve fitting was carried out in SEDPHAT v10.58^51^ using multiple iterations of the Levenberg-Marquardt algorithm for reduction of global χ^2^ value. Heats were fitted to a simple hetero-association model (A + B <-> AB) without a defined stoichiometry (‘n’ value). Incompetent (non-reactive) fractions of K2A1 and VARC were kept as dependent variables in the regression model to account for the uncertainty in deriving ‘n’^52^.

### Microscopy

For immunofluorescence microscopy and co-localization studies, sample slides were prepared and images acquired as previously described^53^. *Plasmodium falciparum* 3D7 infected erythrocytes (iRBCs) synchronized to ring and trophozoite stages were fixed using 4% paraformaldehyde and 0.0075% glutaraldehyde. The iRBCs were probed with rabbit anti-VARC antibodies (1:500) and mouse anti-K2A1 antibodies (1:150) (Bangalore Genei, India). Secondary antibodies used were AlexaFluor488-tagged anti-mouse and AlexaFluor594 tagged anti-rabbit (Invitrogen). Fixed smears were mounted in DAPI Anti-Fade (Invitrogen) and sealed. A smear of uninfected erythrocytes was used as a control.

Co-localization of K2A1 and VARC was visualized on a Nikon A1R confocal laser-scanning microscope equipped with diode (405 nm, DAPI), Ar (488 nm, AlexaFluor488) and He-Ne (594 nm, AlexaFluor594) lasers. Images were acquired separately for each wavelength and merged, using NIS elements v 3.2 software. Quantitation of co-localization was done using Pearson's correlation and co-localization coefficients c1 and c2^31^, calculated by the ‘Colocalization’ module in NIS elements software.

## Author Contributions

N.S.B. designed research; A.K.G., P.R., A.K. and N.S.B. performed research and A.K.G. and N.S.B. analyzed data and wrote the paper.

## Additional information

**Accession codes:** Accession nos. for nucleotide sequences of PfEMP1 and KAHRP are GenBank: XM_001349477.1 and GenBank: XM_001349498.2, respectively. Sequence-specific NMR resonance assignments of K2A1 has been deposited in the BioMagResBank database, www.bmrb.wisc.edu [BMRB accession number 19029].

## Supplementary Material

Supplementary Information Supplementary Dataset 1
