# Supplementary material for: Dynamic association of PfEMP1 and KAHRP in knobs mediates cytoadherence during Plasmodium invasion
Source: Sci Rep. 2015 Mar 2;5:8617. doi: 10.1038/srep08617 (PMC4345318; doi:10.1038/srep08617)
Supplement: Supplementary Information — Supplementary Dataset 1 [file srep08617-s1.pdf]

Dynamic association of PfEMP1 and KAHRP in knobs mediates cytoadherence during Plasmodium invasion

Akshay Kumar Ganguly[1], *Priyatosh Ranjan*[2], *Ashutosh Kumar*[2] *& Neel Sarovar Bhavesh*[1*]

[1]International Centre for Genetic Engineering and Biotechnology (ICGEB), Aruna Asaf Ali Marg, New Delhi, India – 110 067

[2]Department of Biosciences and Bioengineering, Indian Institute of Technology (IIT), Bombay, Mumbai, India – 400 076

[1]To whom correspondence should be addressed. E-mail: neelsb@icgeb.res.in

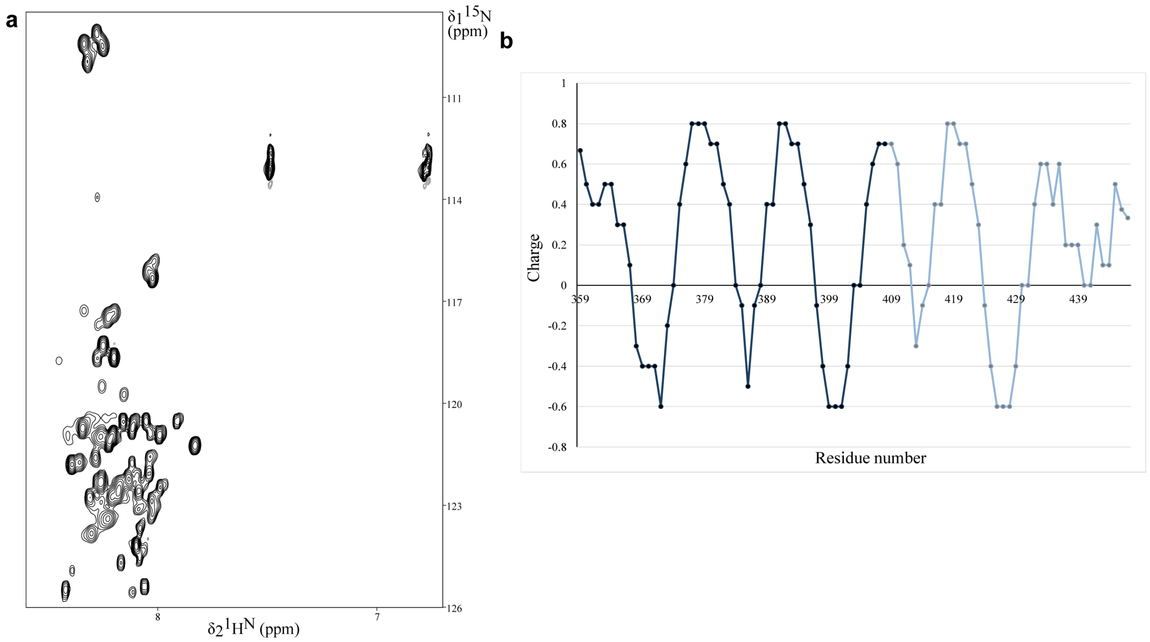

Supplementary figure S1: Low complexity property of K2A and K2A1. (a) [^{15}N, ^{1}H] HSQC spectrum of K2A at 298 K showing poor backbone $^{1}H^{N}$ chemical shift dispersion. **(b)** Sequence specific averaged charge plots (moving window of 5 amino acids) for K2A1 (dark blue) overlaid on that of K2A (light blue).

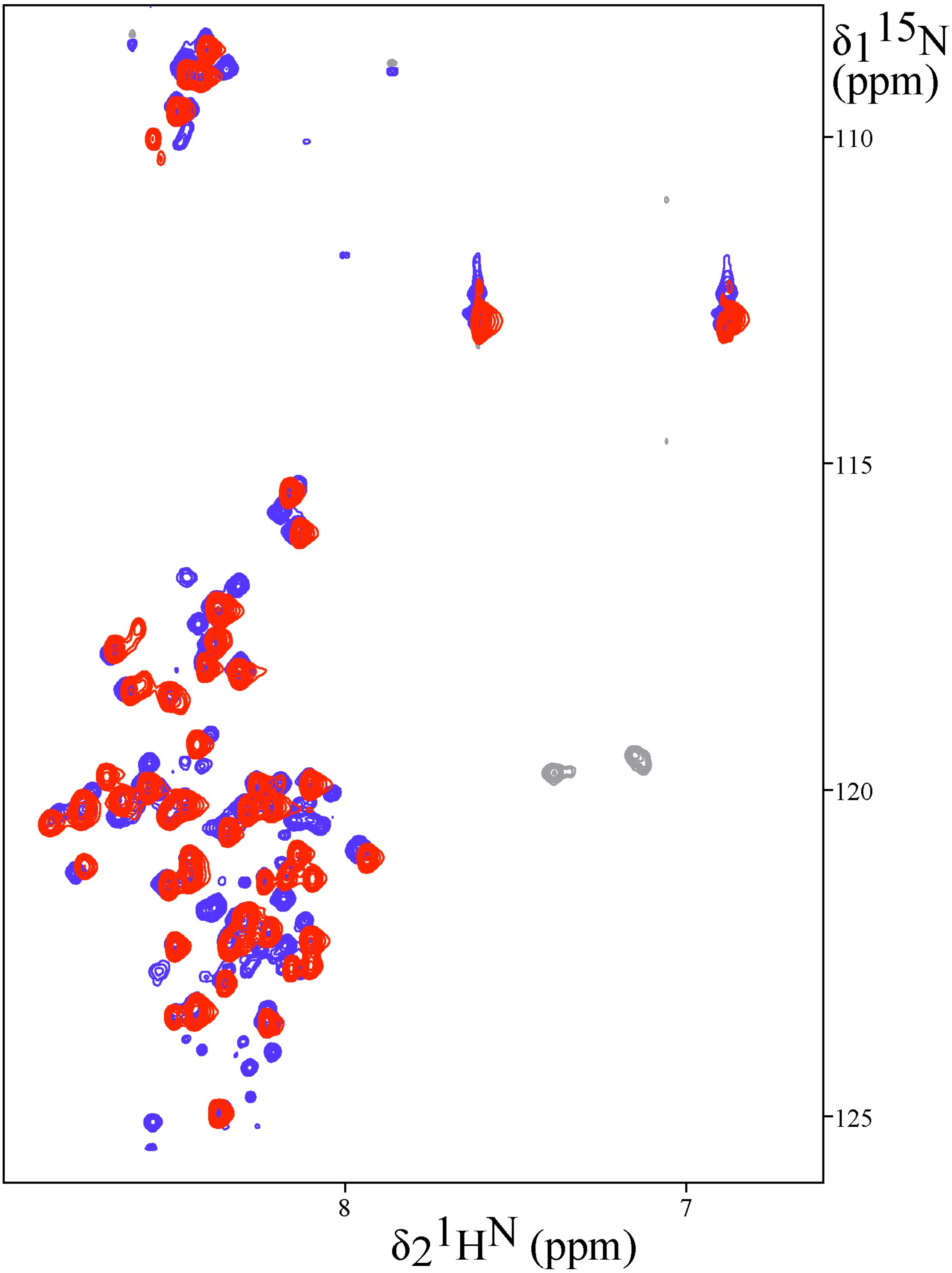

Supplementary figure S2: Spectral overlap of K2A1 over K2A. Overlay of 2D [^{15}N, ^{1}H] HSQC spectra of K2A1 (red) on K2A (blue) at 278 K showing a high number of overlapping resonances.

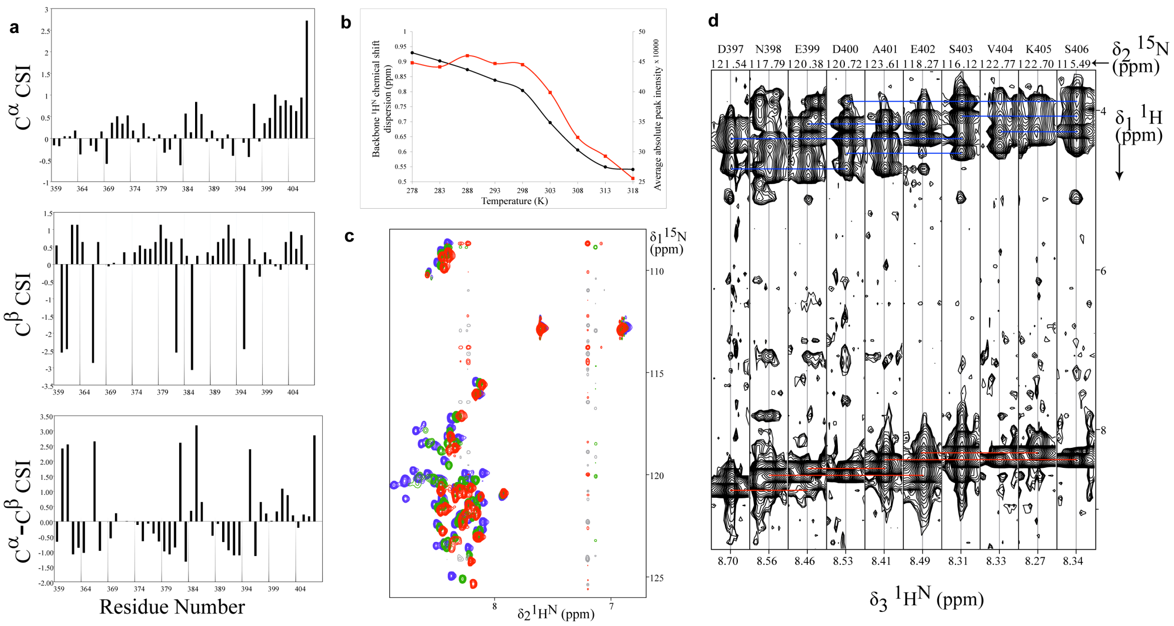

Supplementary figure S3: Effect of temperature on K2A1. (a) Chemical shift indices (CSI) for K2A1 (C^{α}, C^{β}, C^{α}-C^{β}) showing the largely coil-like state of K2A1. **(b)** Changes in backbone $^1H^N$ chemical shift dispersion (black) and average backbone peak intensity (red) upon change in temperature. **(c)** Overlay of 2D [^{15}N, ^1H] HSQC spectra of K2A1 at 278 K (blue), 298 K (green) and 318 K (red). **(d)** 3D ^{15}N-edited [^1H, ^1H] NOESY strips for residues D397 to S406 at 278 K showing short to medium range $^1H^N$ – $^1H^{\alpha}$ (blue) and $^1H^N$ – $^1H^N$ (red) NOE cross peaks.

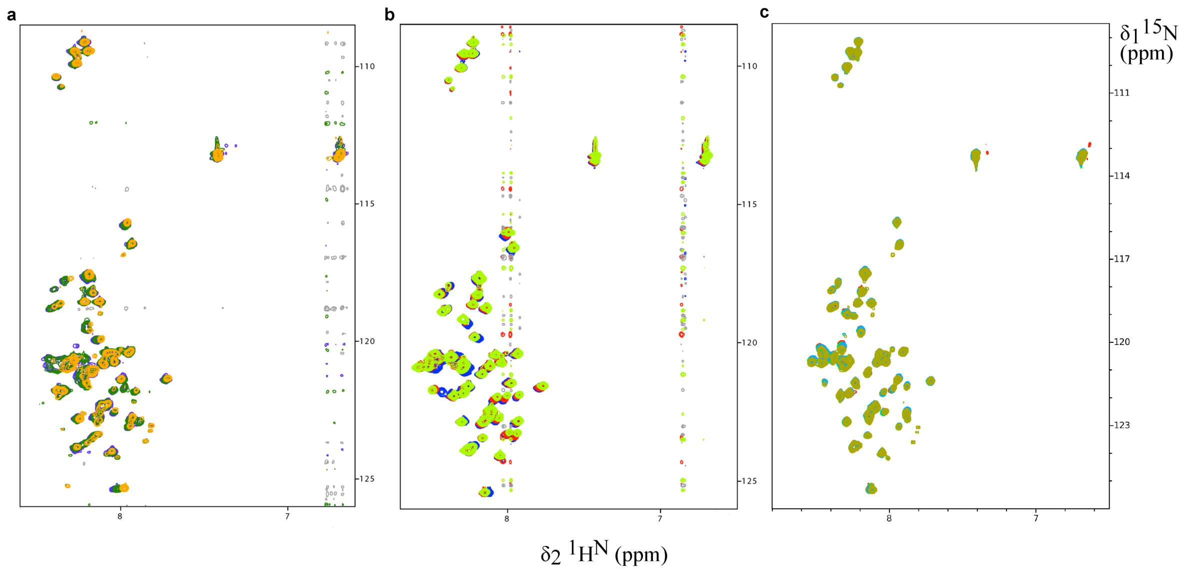

Supplementary figure S4: K2A1 spectra on saturation with VARC. (a) Overlay of 2D [^{15}N, ^1H] HSQC spectra of 1:5 (blue), 1:7.5 (green) and 1:10 (ochre) molar ratios of ^{15}N-labeled K2A1 to unlabeled VARC showing minor changes in resonance frequencies and extensive line broadening. **(b)** Overlay of 2D [^{15}N,^1H] HSQC spectra of 1:5 (green) and 1:1 (red) molar ratios of K2A1 to BSA along with K2A1 alone (blue). **(c)** Overlay of 2D [^{15}N,^1H] HSQC spectra of 1:5 (olive green), 1:2 (blue), 1:1 (cyan) molar ratios of K2A1 to human α-Synuclein along with K2A1 alone (red)

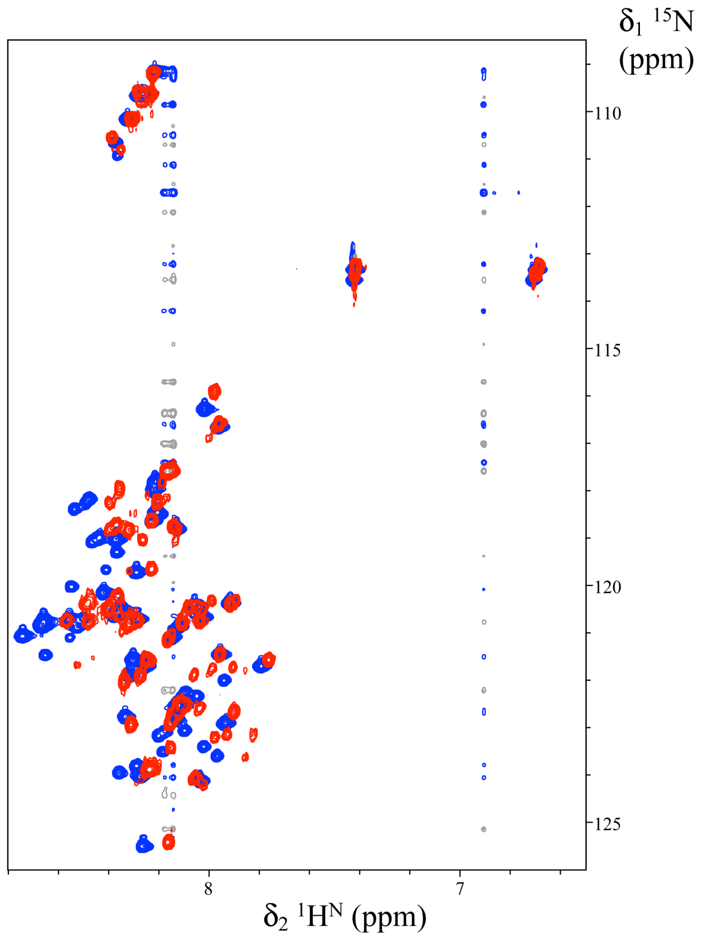

Supplementary figure S5: Effect of NaCl concentration on K2A1 – VARC complex. Overlay of 2D [^{15}N,^{1}H] HSQC spectra of free K2A1 (blue) and K2A1-VARC (1:5 molar ratio) (red) complex at 150 mM NaCl.

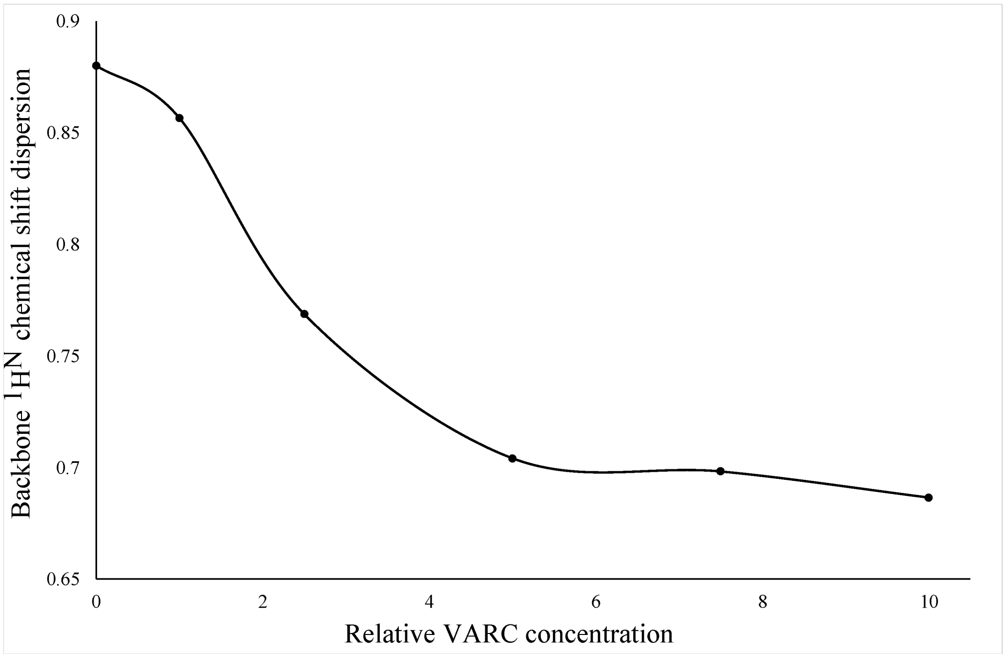

Supplementary figure S6: Conformational shift to coil-like state for K2A1 upon binding VARC. Change in backbone $^{1}H^{N}$ chemical shift dispersion of K2A1 on titrating against increasing concentrations of VARC.

Supplementary table S1: Torsion angle values for residues D397 to S403, approximating to that of a right-handed helix. Values are derived from observed chemical shifts using TALOS+

RESIDUE	ϕ (°)	ψ (°)
D397	-62.376	-32.978
N398	-65.572	-35.746
E399	-65.739	-36.209
D400	-65.965	-38.558
A401	-63.742	-36.223
E402	-70.994	-34.282
S403	-84.409	-22.248

Supplementary table S2: Correlation coefficients between CSP, S^2 and charge for the four interacting regions on K2A1.

Region Number	Residue Range	r(CSP:S^2)[a]	r(CSP:CHARGE)[a]	r(S^2:CHARGE)[a]
1	K359 – D370	-0.6165 (*3.28×10^{-2}*)[b]	0.9349 (*8.25×10^{-6}*)	-0.6306 (*2.79×10^{-2}*)
2	G373 – E388	-0.8601 (*1.93×10^{-5}*)	0.8456 (*3.69×10^{-5}*)	-0.9500 (*2.00×10^{-8}*)
3	K390 – D400	-0.9170 (*7.09×10^{-5}*)	0.9013 (*1.51×10^{-4}*)	-0.9891 (*1.00×10^{-8}*)
4	A401 – K407	0.8866 (*7.82×10^{-3}*)	0.9675 (*3.59×10^{-4}*)	0.8455 (*1.66×10^{-2}*)

a – Pearson's correlation coefficient between variables charge, CSP (chemical shift perturbation) and S^2 (Order parameter).
b - Figures in brackets represent the *P*-value associated with the corresponding correlation coefficient for *n* -2 degrees of freedom

Supplementary table S3: Representative residue level dissociation constants (K_d) from backbone amide chemical shift perturbations.

Residue	K_d (μM)	Statistics	
	Value ± standard error	Reduced χ^2	Adjusted R^2
K378	10 ± 3	0.01	0.96
H382	12 ± 3	0.01	0.94
K390	9 ± 2	0.01	0.96
K405	10 ± 3	0.01	0.96
S406	11 ± 2	0.01	0.96

Supplementary table S4: Co-localization statistics for KAHRP and PfEMP1 (trophozoite stage)

Figure	Pearson's correlation	c1	c2
5b	0.784169	0.979	0.991
5c	0.69507	1	1

c1, c2 – Correlation coefficients of KAHRP (AlexaFluor488) over PfEMP1 (AlexaFluor594) and *vice versa*, respectively
